# Enhancing the Weld Quality of Polylactic Acid Biomedical Materials Using Rotary Friction Welding

**DOI:** 10.3390/polym16070991

**Published:** 2024-04-04

**Authors:** Chil-Chyuan Kuo, Hua-Xhin Liang, Song-Hua Huang, Shih-Feng Tseng

**Affiliations:** 1 Department of Mechanical Engineering, Ming Chi University of Technology, No. 84, Gungjuan Road, New Taipei City 24301, Taiwan; 2 Research Center for Intelligent Medical Devices, Ming Chi University of Technology, No. 84, Gungjuan Road, New Taipei City 24301, Taiwan; 3 Department of Mechanical Engineering, Chang Gung University, No. 259, Wenhua 1st Rd., Guishan Dist., Taoyuan City 33302, Taiwan; 4 Center for Reliability Engineering, Ming Chi University of Technology, No. 84, Gungjuan Road, Taishan District, New Taipei City 24301, Taiwan; 5 Li-Yin Technology Co., Ltd., No. 37, Lane 151, Section 1, Zhongxing Road, Wugu District, New Taipei City 241, Taiwan; 6 Department of Mechanical Engineering, National Taipei University of Technology, No. 1, Sec. 3, Zhongxiao E. Rd., Da’an Dist., Taipei City 106344, Taiwan

**Keywords:** polylactic acid, biomaterial, rotary friction welding, joint strength, high energy efficiency, low environmental pollution

## Abstract

Polylactic acid (PLA) stands out as a biomaterial with immense potential, primarily owing to its innate biodegradability. Conventional methods for manufacturing PLA encompass injection molding or additive manufacturing (AM). Yet, the fabrication of sizable medical devices often necessitates fragmenting them into multiple components for printing, subsequently requiring reassembly to accommodate the constraints posed by the dimensions of the AM platform. Typically, laboratories resort to employing nuts and bolts for the assembly of printed components into expansive medical devices. Nonetheless, this conventional approach of jointing is susceptible to the inherent risk of bolts and nuts loosening or dislodging amid the reciprocating movements inherent to sizable medical apparatus. Hence, investigation into the joining techniques for integrating printed components into expansive medical devices has emerged as a critical focal point within the realm of research. The main objective is to enhance the joint strength of PLA polymer rods using rotary friction welding (RFW). The mean bending strength of welded components, fabricated under seven distinct rotational speeds, surpasses that of the underlying PLA substrate material. The average bending strength improvement rate of welding parts fabricated by RFW with three-stage transformation to 4000 rpm is about 41.94% compared with the average bending strength of PLA base material. The average surface hardness of the weld interface is about 1.25 to 3.80% higher than the average surface hardness of the PLA base material. The average surface hardness of the weld interface performed by RFW with variable rotational speed is higher than the average surface hardness of the weld interface performed at a fixed rotating friction speed. The temperature rise rate and maximum temperature recorded during RFW in the *X*-axis of the CNC turning machine at the outer edge of the welding part surpassed those observed in the internal temperature of the welding part. Remarkably, the proposed method in this study complies with the Sustainable Development Goals due to its high energy efficiency and low environmental pollution.

## 1. Introduction

Rotary friction welding (RFW) is a welding process that joins two pieces of metal by using friction and heat generated by rotating one of the components against the other. This process is known as friction stir welding or inertia friction welding. The features of RFW [1] encompass several advantages, including the absence of electric arcs, low energy consumption [2], and minimal environmental impact [3]. Consequently, RFW finds extensive applications in joining various components [4], such as producing automotive parts, piston rods, shafts, and tubes [5]. RFW is frequently employed to join two cylindrical components, offering numerous benefits such as reduced cycle times, minimized material wastage, superior joint strength, and the capability to bond materials, whether similar or dissimilar. Skowronska et al. [6] examined the structural characteristics of welded joints using high-speed friction welding. The results revealed that the welded joint attained a surface hardness exceeding HV 340. Eliseev et al. [7] explored the microstructural changes occurring in the transfer layer of aluminum (Al) alloy welding parts, uncovering a decrease in grain size and volume fraction toward the center of the layer. Anwar et al. [8] investigated the microstructure of alloy 800H of welding part using RFW after heat treatment, successfully achieving the minimum grain size. Khalaf et al. [9] investigated the heat generation of different tool components. The results showed that the pins with increased edges and a triangular shape demonstrated more pronounced heat generation, influencing the heat flux on polyethylene surfaces. Vidakis et al. [10] examined the welding tool geometry, rotational speed, and travel speed for acrylonitrile butadiene styrene (ABS) fabricated with a material extrusion process [11]. Ma et al. [12] investigated the impact of temperature on the mechanical characteristics of aluminum alloy joints via friction stir welding. The results unveiled a reduced gradient across the thickness, heightened heat input, and amplified material flow at the base. Notably, the welded components showcased superior mechanical prowess when juxtaposed with three-dimensional printed counterparts of congruent geometry. Yang et al. [13] delved into temperature characterization and contact dynamics during welding, employing the harmonic balance method. Both simulation and experimental results underscored the significant impact wielded by welding duration and amplitude on the interface temperature. Maggiore et al. [14] conducted an extensive review focusing on structural adhesive joints within hybrid joining methodologies. Their findings advocate the potential of hybrid joining technologies as a versatile solution applicable across various industries, aiming at mitigating manufacturing burdens and expenses. Pereira et al. [15] examined the impact of various welding parameters on the mechanical strength of welding parts using friction stir welding with polymers. The results suggested that an elevated rotational speed/welding speed ratio improved joint efficiency.

Polylactic acid (PLA) [16], a biodegradable thermoplastic, originates from renewable sources, predominantly extracted from corn starch. Classified as a polyester, PLA serves as a prominent bioplastic in a myriad of applications [17]. It has gained popularity in recent years as a more environmentally friendly alternative to traditional petroleum-based plastics [18,19,20]. PLA has various applications across various industries due to its biodegradability, renewability, and versatility. Some common applications of PLA include medical devices [21], dental implants [22,23], and food service products [24,25]. When constrained by the dimensions of the additive manufacturing platform [26], it is common practice to divide a substantial medical device into multiple segments for printing, necessitating subsequent assembly.

Commonly, laboratories extensively utilize nuts [27] or bolts [28] for amalgamating three-dimensional (3D)-printed components into sizable physical models. Nonetheless, this conventional joining method is prone to bolts and nuts loosening or detachment during the reciprocating motion inherent to large physical models. Furthermore, the junctions formed by bolts and nuts may induce micro-cracks in the structure of large physical models, potentially leading to consequential damage. Consequently, the exploration of joining technologies for integrating 3D-printed parts into expansive physical models has emerged as a crucial research avenue. The main objective is to improve the weld quality of PLA polymer rods using RFW [29,30]. Following the RFW process, bending tests [31] and Shore A surface hardness tests [32] were conducted to analyze the mechanical properties of the welded components. The temperature histories of the three temperature measurement locations in both the *X*-axis and *Z*-axis of the CNC turning machine were also investigated during RFW with seven rotational speeds using both thermocouples and an infrared camera. Finally, a comprehensive technical database was established for RFW using PLA biomedical material.

## 2. Experimental Details

**Figure 1:**
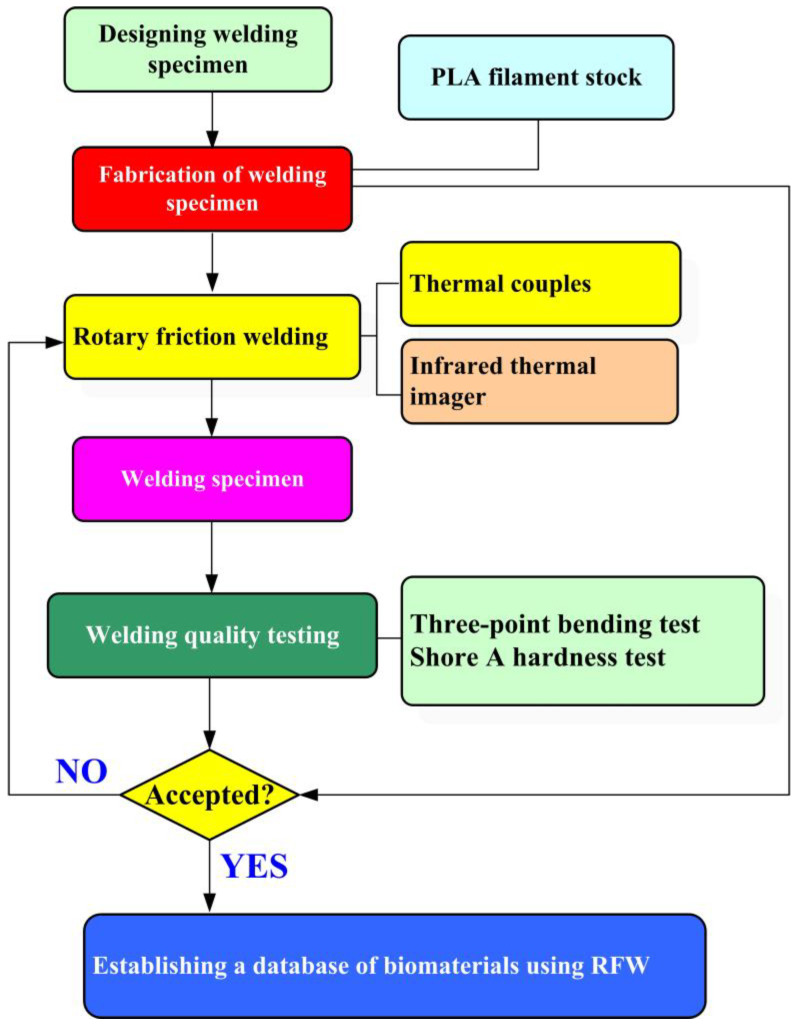
Research process in this study.

**Figure 2:**
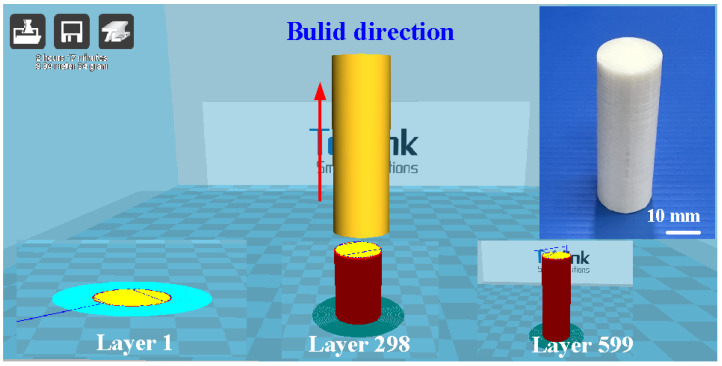
Slicing results of the PLA biomedical polymer rod in the slicing software.

**Figure 3:**
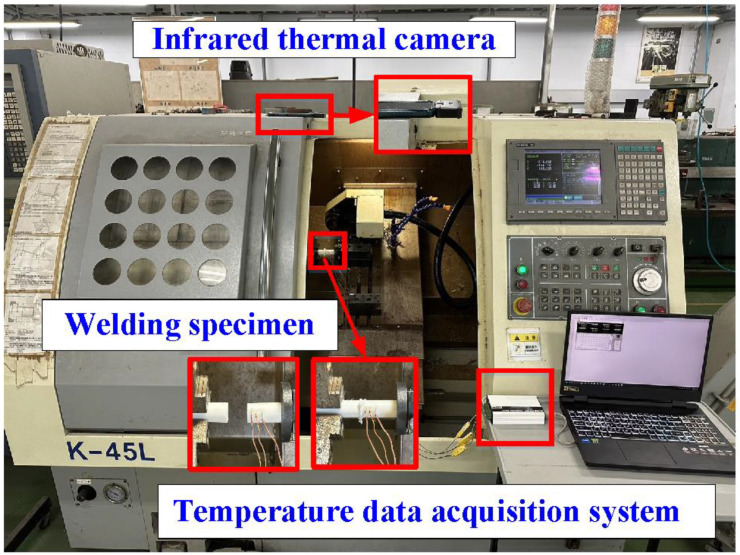
Experimental configuration devised for gauging the welding temperature of PLA biomedical polymer rods during RFW.

Figure 1 depicts the research process of this study. In this study, a PLA filament stock (Thunder 3D Inc., New Taipei City, Taiwan) was used to print welding workpieces using a fused filament fabrication machine (Teklink solution Inc., New Taipei City, Taiwan). Subsequent to the RFW process, the bending strength of the welding components was evaluated utilizing a three-point bending test machine (RH-30, Shimadzu Inc., Kyoto, Japan) set at a movement speed of around 1 mm/s. Additionally, the assessment of weldment quality involved the application of the Shore A surface hardness test (MET-HG-A, SEAT Inc., New Taipei City, Taiwan) and RH-30 equipment (Shimadzu Inc., Kyoto, Japan). The fracture surfaces were further analyzed using optical microscopy (OM) (Quick Vision 404, Mitutoyo Inc., Tokyo, Japan). Finally, a comprehensive technical database was established for the characterization of PLA weldments fabricated by RFW. Figure 2 shows the slicing results of the PLA biomedical polymer rod in slicing software (UltiMaker Cura, Utrecht, The Netherlands). The welding specimen features a cylindrical cross-section with dimensions of approximately 20 mm in diameter and 40 mm in length. Figure 3 depicts the experimental configuration devised for gauging the welding temperature of PLA biomedical polymer rods during RFW. A computerized turning machine (K-45L, Kae Jiuh, Inc., New Taipei City, Taiwan) was employed to join the PLA biomedical polymer rods. The process commenced by securing one workpiece using a chuck and subjecting it to a continuous rotational motion, while the other workpiece remained firmly stationary. In the process of RFW, friction heat was generated at the interface of the two welding specimens. The welding specimens were subjected to pressure until the weld joint was successfully formed. To further improve the mechanical properties of welded parts, an autotransformer was set to 50 V for ultrasonic transduction (WB 2835-45HB, Whirl Best International, Inc., New Taipei City, Taiwan). The frequency and amplitude of the ultrasonic transducer were 28 KHz and 0.013 mm, respectively. A load cell (ARI742, Zhiheng Industrial Co., Inc., New Taipei City, Taiwan) was employed to measure the welding force during RFW. During the RFW process, the temperature profiles at both the weld interface and within the welding specimens were recorded with k-type thermocouples (C071009-079, Cheng Tay Inc., New Taipei City, Taiwan) and an infrared camera (BI-TM-F01P, Panrico Trading Inc., New Taipei City, Taiwan). Additionally, a data acquisition system (MRD-8002L, IDEA System Inc., New Taipei City, Taiwan) was utilized for collecting temperature change data.

**Figure 4:**
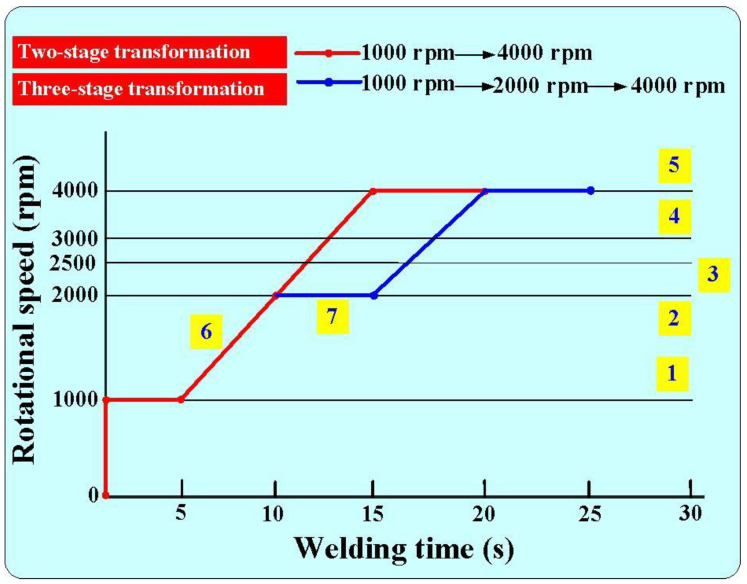
Seven rotational speeds used in the RFW.

**Figure 5:**
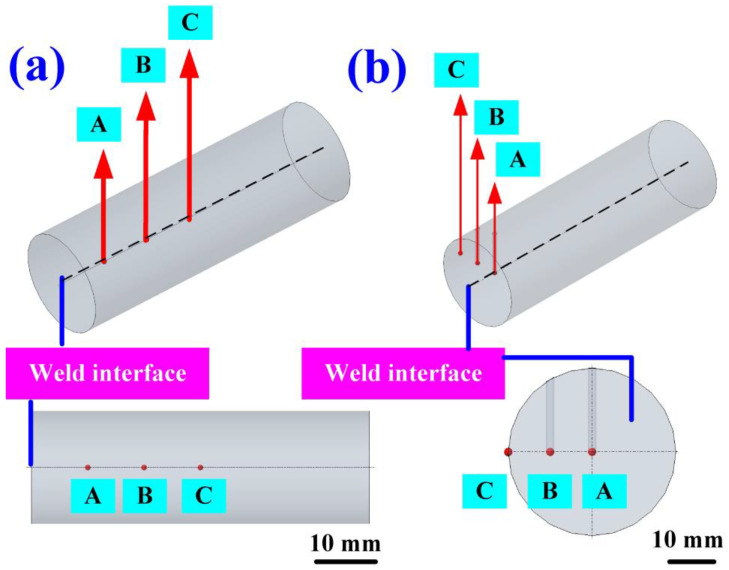
Schematic diagram of three temperature measurement locations in the (**a**) *Z*-axis and (**b**) *X*-axis directions of the CNC turning machine.

Figure 4 shows the seven rotational speeds used in this study. The process parameters of RFW include an axial load of 24.1 N, welding pressure of 0.077 MPa, burn-off length of 2 mm, and feed rate of 6 mm/min. It is noteworthy that the rotational speeds denoted as 1, 2, 3, 4, and 5 maintained a consistent value. Specifically, these rotational speeds correspond to 1000 rpm, 2000 rpm, 2500 rpm, 3000 rpm, and 4000 rpm, respectively. The RFW cycle duration with the constant rotational speed spans 90 s, comprising 30 s of friction time, 30 s of welding time, and 30 s of cooling time. Number 6 underwent a two-stage transformation to reach 4000 rpm. In the first stage, it accelerated from a standstill to 1000 rpm. In the second stage, it accelerated from 1000 rpm to 4000 rpm. The cycle time for the RFW with the two-stage transformation to 4000 rpm was 80 s, comprising a friction time of 30 s, a welding time of 20 s, and a cooling time under pressure of 30 s. Number 7 underwent a three-stage transformation to reach 4000 rpm. The first stage involved accelerating from a standstill to 1000 rpm. The second stage accelerated from 1000 rpm to 2000 rpm, and the third stage accelerated from 2000 rpm to 4000 rpm. The cycle duration for RFW featuring a three-stage transformation up to 4000 rpm totaled 85 s. This encompasses 30 s of friction time, followed by 25 s of welding time, and concludes with 30 s of cooling time under pressure. Figure 5 shows the schematic diagram of the three temperature measurement locations in the *Z*-axis and *X*-axis directions of the CNC turning machine. In the *Z*-axis, the distances between points A, B, and C and the weld interface measure 1 mm, 2 mm, and 3 mm, respectively. Along the *X*-axis, point A was positioned at the center of the welding specimen. The distances between points A, B, and C, and the center of the welding specimen amounted to approximately 0 mm, 5 mm, and 10 mm, respectively.

## 3. Results and Discussion

**Figure 6:**
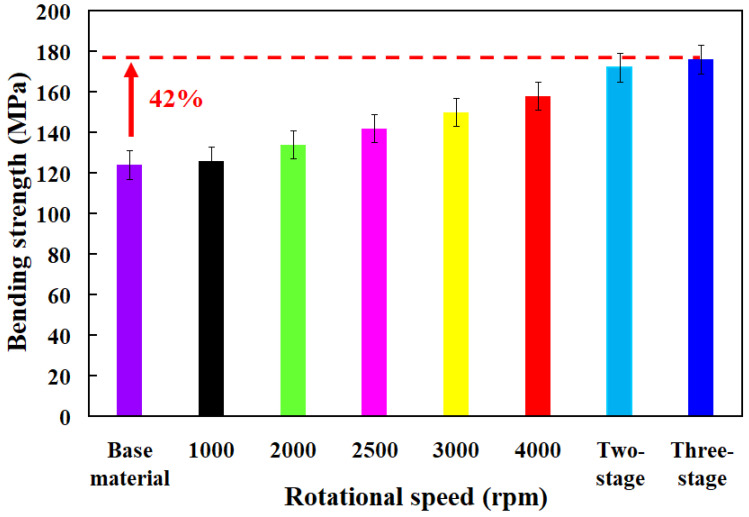
Bending strength of the base material and welding parts fabricated by RFW with seven rotational speeds.

**Figure 7:**
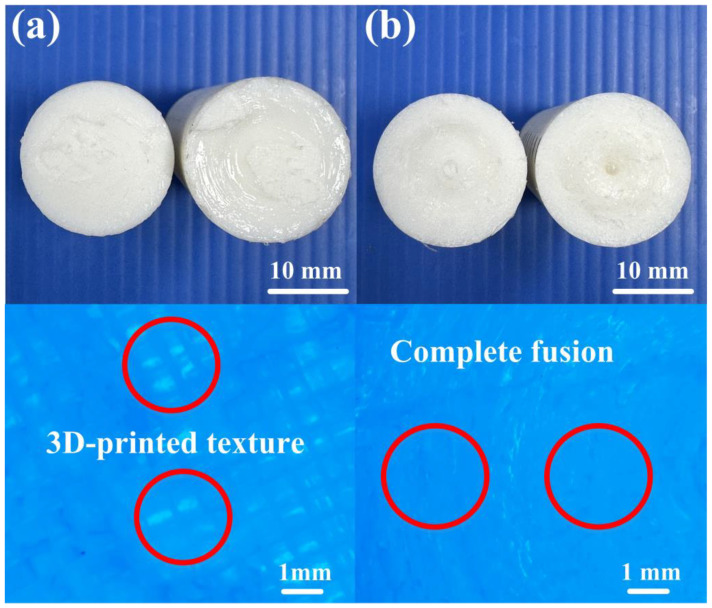
Fracture surfaces of the specimens fabricated by (**a**) 3D printing and (**b**) welding parts fabricated by RFW with three-stage transformation after the bending test.

**Figure 8:**
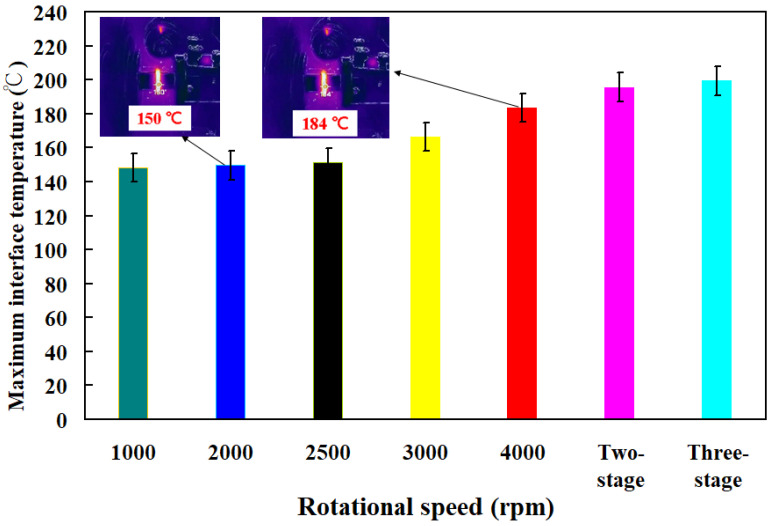
Maximum interface temperature of welding parts fabricated by RFW with seven rotational speeds.

In this study, five specimens were examined. Figure 6 shows the bending strength of the base material and welding parts fabricated by RFW with seven rotational speeds. Figure 7 shows the fracture surfaces of the specimens fabricated by 3D printing and welding parts fabricated by RFW with three-stage transformation after the bending test. Three-dimensional printing employs layer-by-layer processing. After conducting bending tests, it was evident that the 3D-printed texture of the welding specimen appeared on the fractured surface. These 3D-printed parts underwent a three-stage transformation RFW. Interestingly, the 3D-printed texture was no longer visible on the fractured surface after the bending test, indicating a complete fusion of weld interface materials. Consequently, the bending strength of welded parts significantly surpassed that of the base materials. The results showed two phenomena. One is that the average bending strength of the welding parts using seven rotational speeds was higher than that of the PLA base material [33]. The average bending strengths of welding parts using seven rotational friction welding speeds were about 134 MPa, 142 MPa, 150 MPa, 158 MPa, 172 MPa, and 176 MPa, respectively. The other is that the average bending strength improvement rates of welding parts using seven rotational friction welding speeds were approximately 1.61%, 8.06%, 14.52%, 20.97%, 27.42%, 38.71%, and 41.94% compared with the average bending strength of PLA base material. Figure 8 shows the maximum interface temperature of welding parts fabricated by RFW with seven rotational speeds. The results showed that the maximum temperature of the weld joint increased accordingly. The maximum temperatures of the weld joint were approximately 148 °C, 150 °C, 151 °C, 166 °C, 184 °C, 196 °C, and 199 °C for seven rotational speeds.

**Figure 9:**
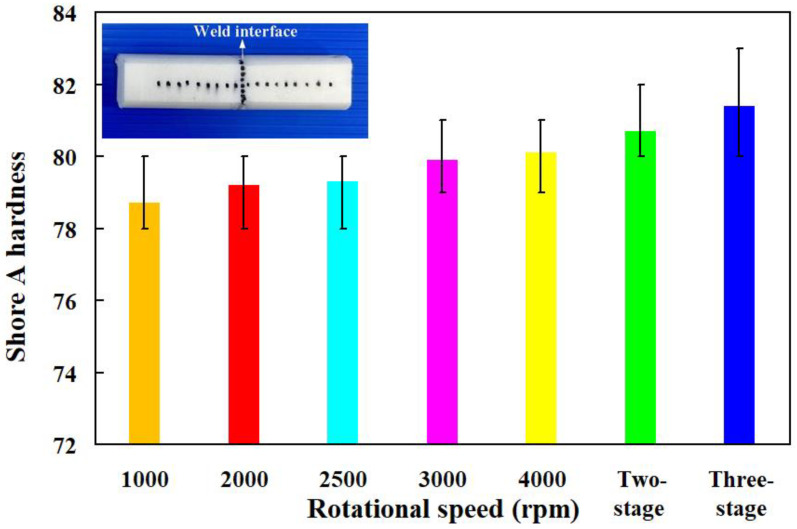
Average Shore A surface hardness in the weld interface of welding parts fabricated by RFW with seven rotational speeds.

**Figure 10:**
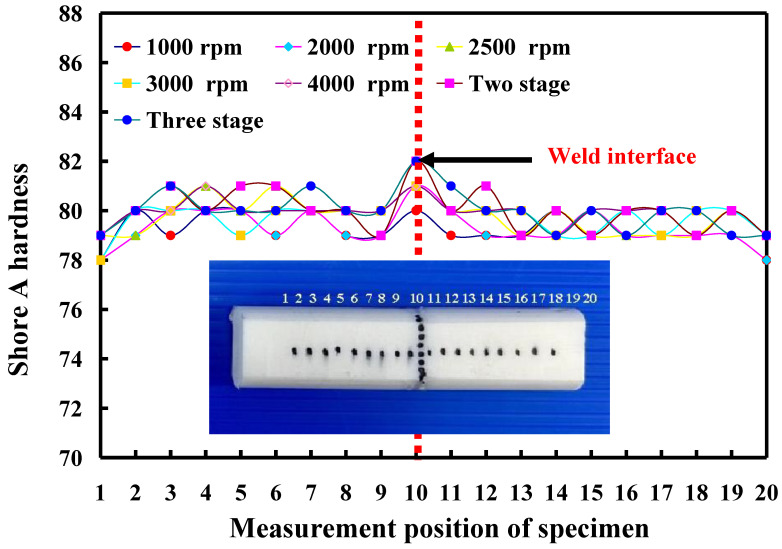
Shore A surface hardness distributions of the welding parts fabricated by RFW with seven rotational speeds.

The evaluation of Shore A surface hardness involves the assessment of material hardness through a universally adopted scale. This evaluation process facilitates the identification of appropriate materials tailored to distinct uses, thereby guaranteeing that welding specimens adhere to criteria for endurance and operational efficacy. In this experiment, five specimens were examined. There were ten measurement points in the weld interface of the welded part, while the surface hardness distributions of the welded part were measured at twenty points. Figure 9 shows the average Shore A surface hardness in the weld interface of welding parts fabricated by RFW with seven rotational speeds. In this study, two specimens were analyzed. Figure 10 shows the shore A surface hardness distributions of the welding parts fabricated by RFW with seven rotational speeds. The results showed that the average surface hardnesses of the seven RFW methods were approximately HS 78.7, HS 79.2, HS 79.3, HS 79.9, HS 80.1, HS 80.7, and HS 81.4, respectively. Based on these results, this study found three phenomena. One is that the average surface hardness of the weld interface is higher than the average surface hardness of the PLA base material. The average surface hardness increases by about 1.27%, 2.53%, 1.25%, 1.25%, 1.25%, 3.80%, and 2.5%, respectively. Secondly, the average surface hardness of the weld interface performed by RFW with variable rotational speed is higher than the average surface hardness of the weld interface performed at a fixed rotating friction speed. The other is that the average surface hardness of the weld interface performed by RFW with three-stage transformation to 4000 rpm is the highest, followed by the average surface hardness of the welding interface performed by RFW with two-stage transformation to 4000 rpm.

**Figure 11:**
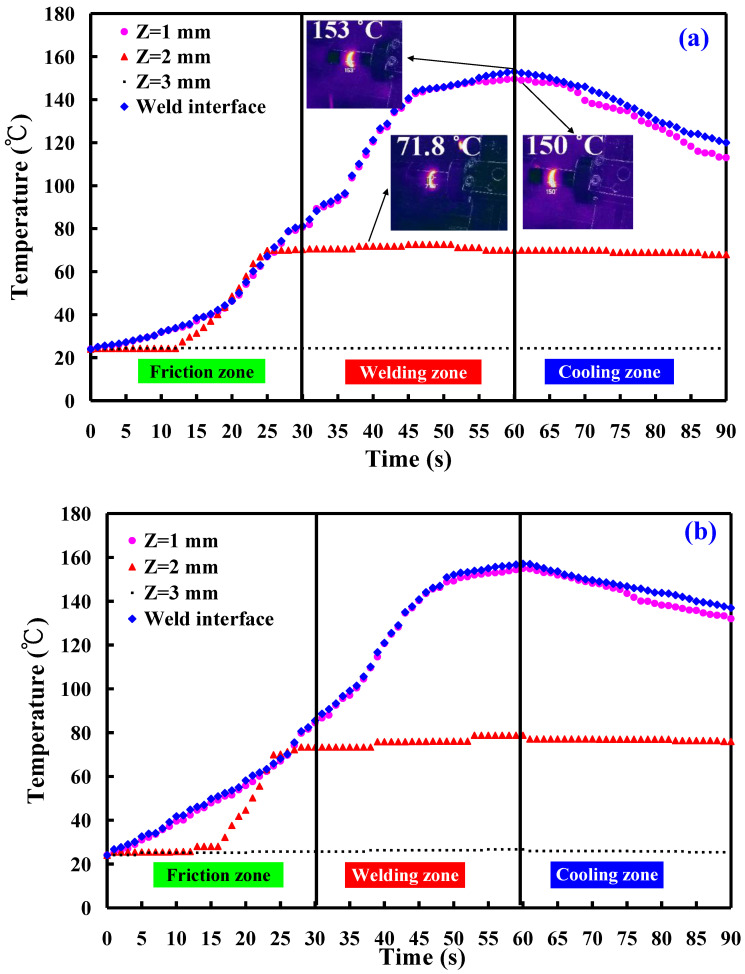

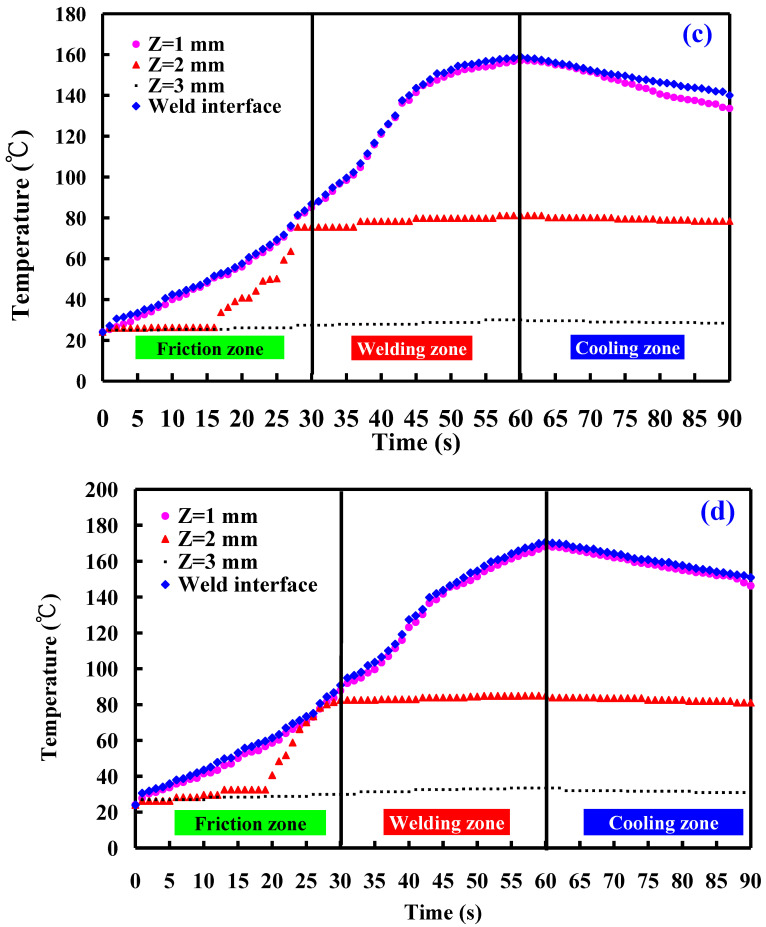

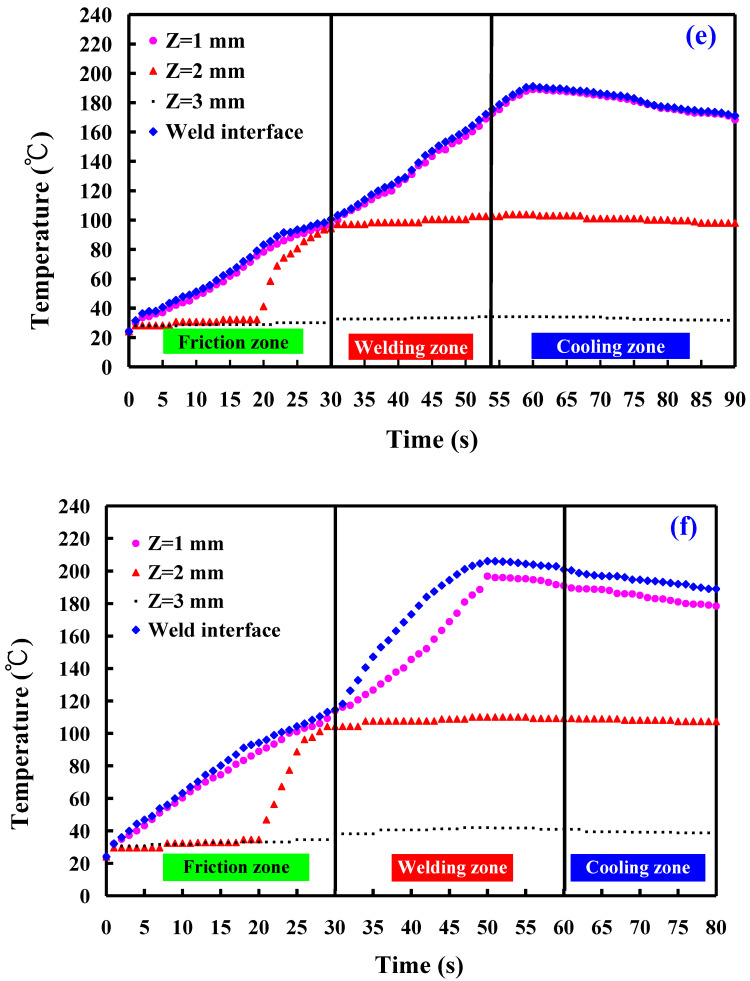

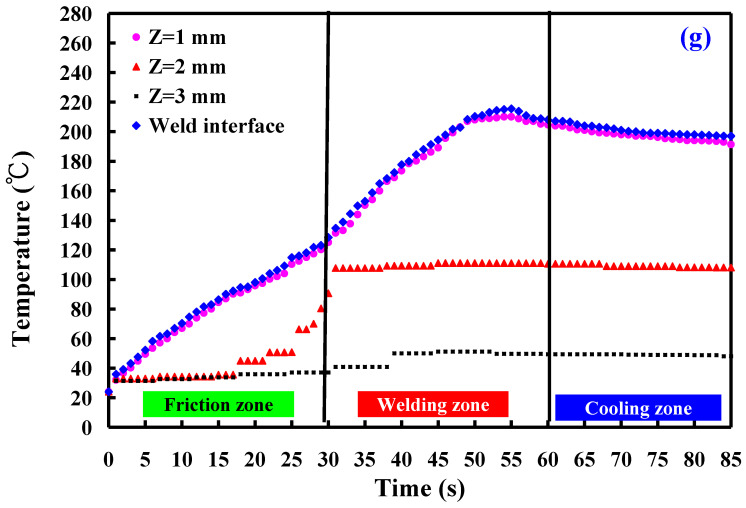
Temperature histories of the three temperature measurement locations in the *Z*-axis of the CNC turning machine: (**a**) 1000 rpm, (**b**)2000 rpm, (**c**) 2500 rpm, (**d**) 3000 rpm, (**e**) 4000, (**f**) two-stage transformation to 4000 rpm, and (**g**) three-stage transformation of the rotational speed to 4000 rpm.

**Figure 12:**
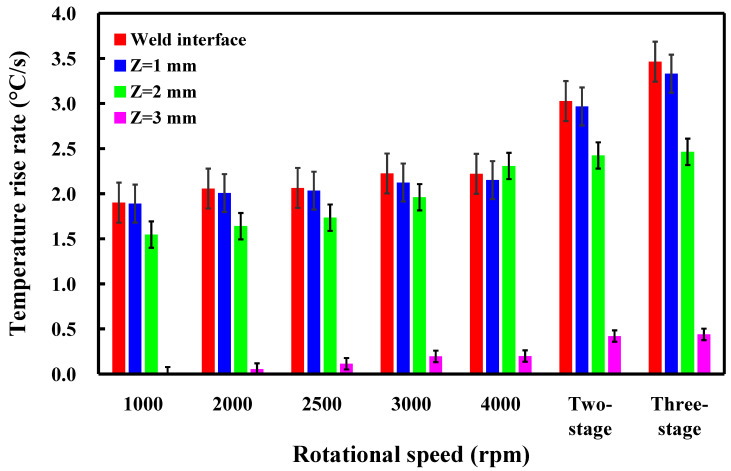
Temperature rise rate during RFW in the *Z*-axis of the CNC turning machine using seven rotational speeds.

**Figure 13:**
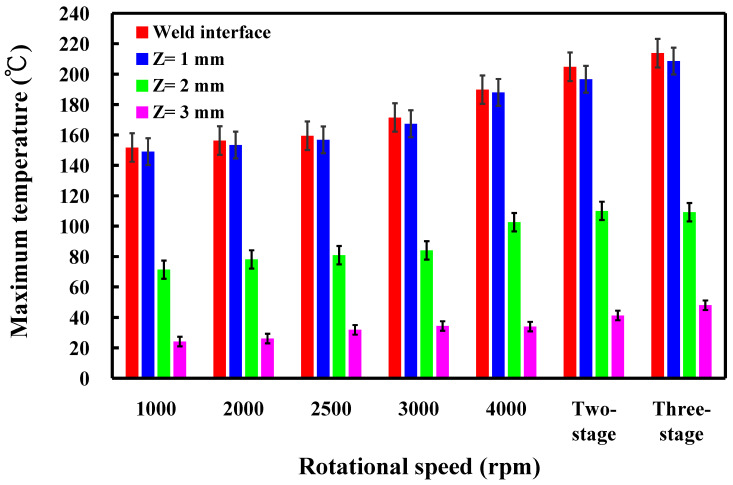
Maximum temperatures measured during RFW in the *Z*-axis of the CNC turning machine using seven rotational speeds.

This study involved the examination of five specimens. Figure 11 shows the temperature histories of the three temperature measurement locations in the *Z*-axis of the CNC turning machine for seven rotational speeds. Figure 12 shows the temperature rise rate during RFW in the *Z*-axis of the CNC turning machine using seven rotational speeds. Figure 13 shows the maximum temperatures measured during RFW in the *Z*-axis of the CNC turning machine using seven rotational speeds. Based on the above results, this study found three phenomena. First, the temperature histories of the three measurement points A, B, and C are not similar. Second, the temperature rise rate of the weld interface does not change significantly when the rotational speed is constant. However, the temperature rise rate of the weld interface shows some changes for a two-stage transformation to 4000 rpm and a three-stage transformation to 4000 rpm. The temperature rise rate of the weld interface is higher than that of the weld interface using fixed rotational speed. Third, the maximum temperatures measured during RFW gradually increase as the rotational speed increases. However, the maximum temperatures measured during RFW do not change significantly for two-stage transformation to 4000 rpm and three-stage transformation to 4000 rpm.

**Figure 14:**
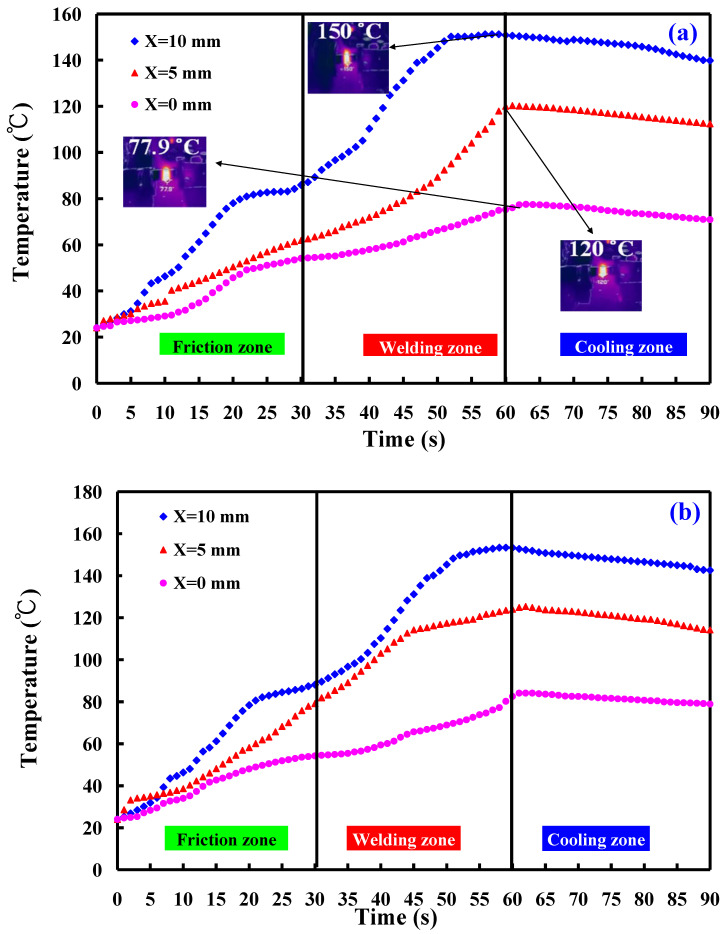

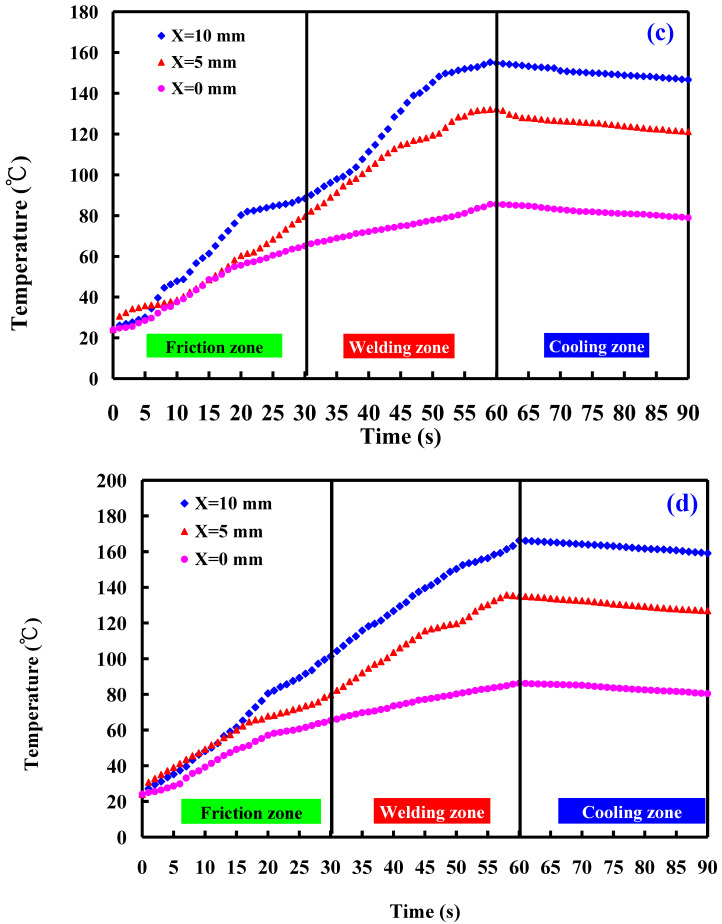

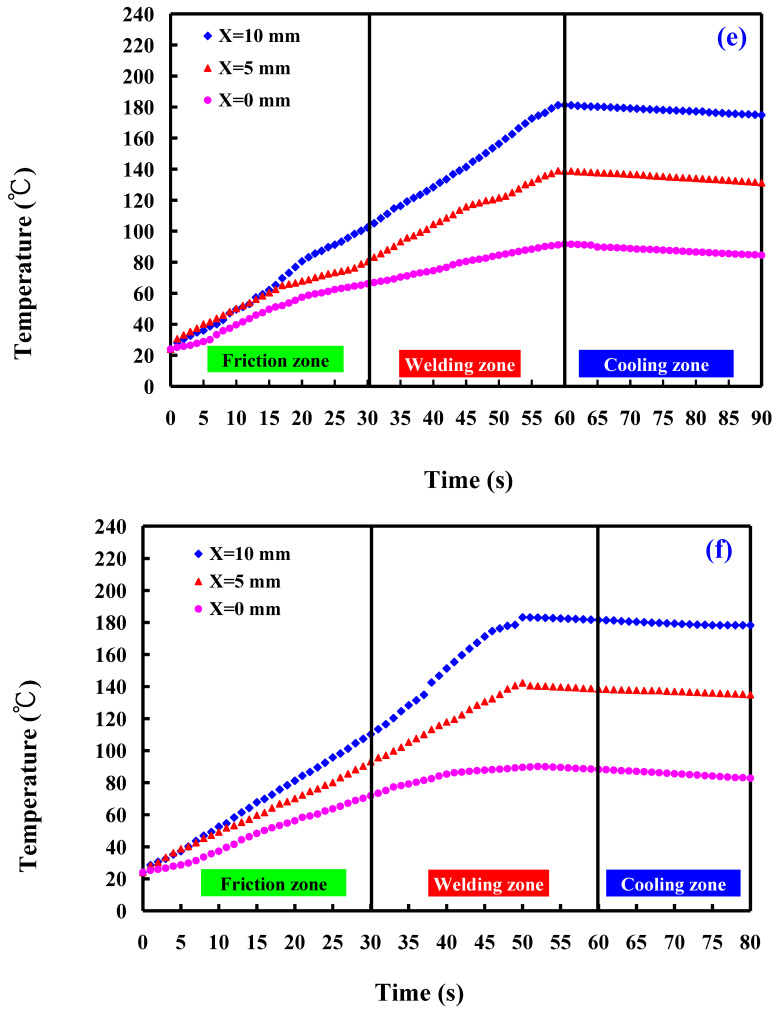

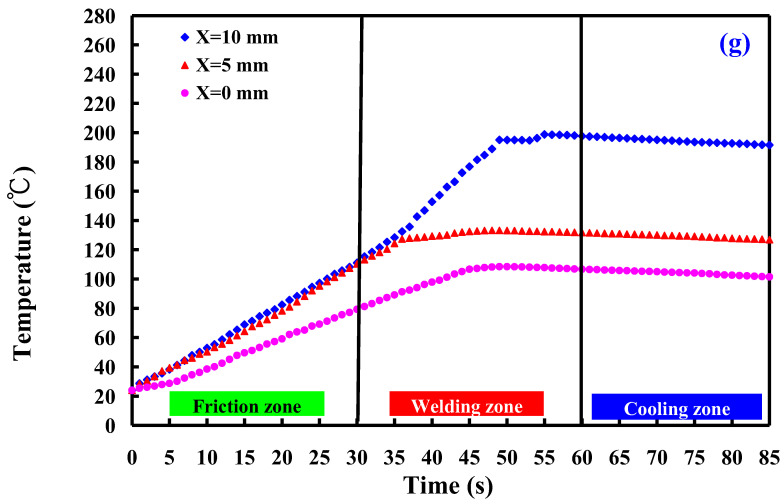
Temperature histories of the three temperature measurement locations in the *X*-axis of the CNC turning machine: (**a**) 1000 rpm, (**b**) 2000 rpm, (**c**) 2500 rpm, (**d**) 3000 rpm, (**e**) 4000, (**f**) two-stage transformation to 4000 rpm, and (**g**) three-stage transformation of the rotational speed to 4000 rpm.

**Figure 15:**
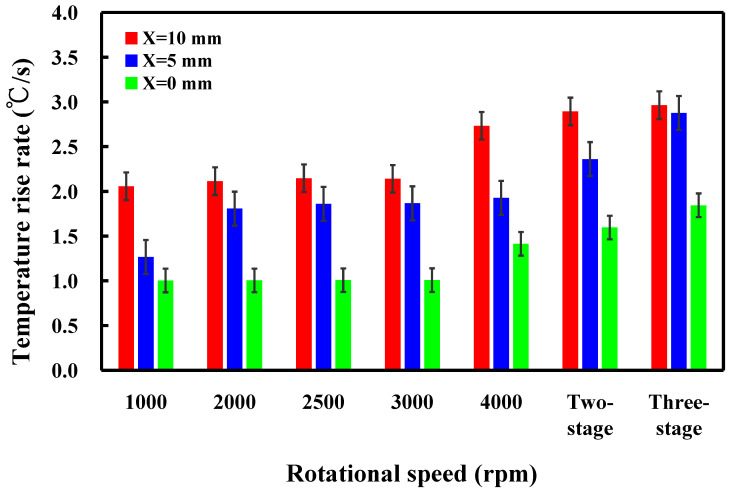
Temperature rise rate during RFW in the *X*-axis of the CNC turning machine using seven rotational speeds.

**Figure 16:**
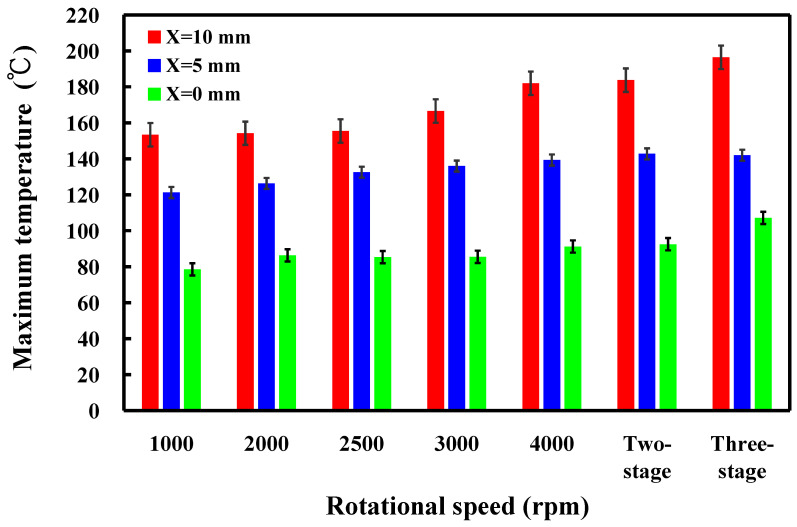
Maximum temperatures measured during RFW in the *X*-axis of the CNC turning machine using seven rotational speeds.

This study involved the examination of five specimens. Figure 14 shows the temperature histories of the three temperature measurement locations in the *X*-axis of the CNC turning machine. Figure 15 shows the temperature rise rate during RFW in the *X*-axis of the CNC turning machine using seven rotational speeds. Figure 16 shows the maximum temperatures measured during RFW in the *X*-axis of the CNC turning machine using seven rotational speeds. Differential temperature profiles were observed among the three measurement points, A, B, and C, attributable to the variation in linear velocity across the welding part. Specifically, the linear velocity at the outer edge of the welding part exceeded that of the internal regions, contributing to distinct temperature histories at these points. Consequently, both the temperature rise rate and the maximum temperature recorded during RFW at the outer edge surpassed those observed internally within the welding part. According to the existing literature, the thermal conductivity of PLA material at room temperature ranges between 0.13 and 0.183 W/m-K [34,35,36]. In this study, the heat source generated by the rotating friction welding interface facilitated temperature measurements at three points: A, B, and C. Notably, the determined thermal conductivity of PLA materials in this study approximated 0.179 W/m-K. This result is generally consistent with the data in the literature. This result also proves that the temperature data measured in this work are correct.

PLA presents an environmentally friendly substitute for conventional plastics, sourced from renewable reservoirs such as corn starch or sugarcane. Its notable characteristics encompass eco-degradation, renewable derivation, adaptability, lucidity, and minimal toxicity. PLA can decompose under specific circumstances, aiding in curbing plastic waste and reducing reliance on fossil fuels. In general, the FRW exhibits lower energy consumption than conventional gas arc welding [37]. Consequently, the outcomes of this study bear practical significance for industrial applications and align with Sustainable Development Goals 7, 9, 10, and 12 [38]. Previous research has delved into evaluating the fatigue life of welded components through fatigue experiments [39] and conducting numerical investigations [40] to explore the optimal process parameters for RFW. Developing numerical models to simulate the RFW process [41,42,43] can aid in predicting and optimizing welding outcomes and understanding the thermal and mechanical aspects of the process. Optimizing the critical process parameters of RFW to enhance efficiency, quality, and cost-effectiveness is an important research topic. In this study, bending tests and Shore A surface hardness tests were performed to investigate the mechanical properties of the welded components. However, the thermal properties of the welded materials were not investigated. Thus, the thermal transitions of the welding parts can be examined by differential scanning calorimetry in future work [44,45]. Furthermore, conducting an in-depth microstructural analysis of the welded joints to understand the effects of the welding process on the material properties [46] using scanning electron microscopy [47] is also an important research topic. These intriguing research topics are currently under exploration.

## 4. Conclusions

The main objective of this work is to enhance the joint strength of PLA polymer rods built with additive manufacturing using RFW. This proposes an approach for the RFW of PLA polymeric rods by varying the rotational speed. After FRW, the mechanical properties of the welding parts were examined by the Shore A surface hardness test, thermal analysis, and three-point bending test. The main conclusions from the experimental work in this study are as follows:

- The average bending strength of the welded components utilizing seven distinct rotational speeds surpasses that of the PLA base material.
- The average bending strength enhancement rate for welded components produced through RFW with a three-stage transformation at 4000 rpm is approximately 41.94% when compared to the average bending strength of the PLA base material.
- The average surface hardness at the weld interface achieved through RFW with variable rotational speed exceeds that of the weld interface produced by a fixed rotating friction speed. The average surface hardness at the weld interface is approximately 1.25% to 3.80% higher than the average surface hardness of the PLA base material.
- The temperature rise rate in the *Z*-axis of the CNC turning machine does not change significantly when the rotational speed is constant. The maximum temperatures measured during RFW gradually increase as the rotational speed increases. Both the temperature rise rate and maximum temperature measured during RFW in the *X*-axis of the CNC turning machine at the outer edge of the welding part are higher than that of the internal temperature of the welding part.
- The thermal conductivity of PLA materials is about 0.179 W/m-K, which is generally consistent with the data in the literature.

## Data Availability

Data and materials are available.
